# Editorial: Insights in systems microbiology: 2021

**DOI:** 10.3389/fmicb.2022.988296

**Published:** 2022-08-25

**Authors:** Qi Zhao, George Tsiamis

**Affiliations:** ^1^School of Computer Science and Software Engineering, University of Science and Technology Liaoning, Anshan, China; ^2^Department of Environmental Engineering, University of Patras, Agrinio, Greece

**Keywords:** systems microbiology, multidisciplinary approaches, gut microbiota, drug-microbe association, computational methods

The aim of systems microbiology is to understand life's circuit diagrams and gain knowledge about the relationships between the individual components that build a cellular organism, a community, and an entire ecological niche (Vieites et al., 2009). That means systems microbiology tends to treat the community as a whole, integrating multidisciplinary approaches to finally make a big picture of how a microbial cell or community operates. We are now entering the third decade of the twenty-first century, and, especially in the last years, the achievements made by scientists in the field of microbiology have been exceptional, leading to significant advancements. This Research Topic collects new insights, novel developments, current challenges, latest discoveries, recent advances, and future perspectives in the field of systems microbiology.

We are pleased to note that our Research Topic has attracted contributions from many highly regarded researchers in this field around the world, including from China, the USA, Italy, and Singapore. We received 15 submissions, 10 of which were accepted for publication after rigorous reviews.

In this special issue, three articles were focused on the new findings in gut microbiota. For example, Zhu Y. et al. proposed a new perspective of intestinal microbiota–neural mitochondria interaction as a communicating channel from gut to brain. Such research could help to extend the vision of gut-brain axis regulation and provide additional research directions on the treatment and prevention of responsive neurological disorders. Accumulating evidence is focused on the roles of the gut microbial community in cardiovascular disease, but few studies have unveiled the alterations and further directions of gut microbiota in severe chronic heart failure (CHF) patients. To investigate this deficiency, Sun et al. collected fecal samples from 29 CHF patients diagnosed with NYHA Class III-IV and 30 healthy controls and then analyzed them using bacterial 16S rRNA gene sequencing. As a result, there were many significant differences between the two groups. Moreover, gut microbiome-based therapeutics have shown promise in ameliorating chronic inflammation. However, they are largely experimental, context- or strain-dependent, and lack a clear mechanistic basis. In this perspective, Koduru et al. reasoned that the future transition toward precision probiotics thus lies in deciphering ligand-receptor interactions, with aryl hydrocarbon receptor (AhR) being a key mediator in managing chronic inflammation.

Three research papers were included in the Research Topic to directly investigate the drug-microbe associations, surfaced enhanced Raman spectra (SERS), and the integration of “multiomic” data with other omics through artificial intelligence algorithms. Zhu B. et al. designed a deep learning-based model named Nearest Neighbor Attention Network (NNAN). The proposed model consists of four components: a similarity network constructor, a nearest-neighbor aggregator, a feature attention block, and a predictor. Under both a cross-validation setting and a realistic potential linkage discovery setting, the empirical comparison of the proposed framework with three state-of-the-art baselines demonstrates that NNAN has significant competitive performance in predicting drug–microbe associations. In another study, Tang et al. used SERS combined with unsupervised and supervised machine learning algorithms to detect 15 bacterial pathogens from clinical samples. According to the results, SERS could accurately identify bacterial pathogens at a general level with comparatively high specificity and sensitivity through the assistance of machine learning methods. Moreover, artificial intelligence (AI) and machine learning (ML) algorithms coupled with other multiomics data (i.e., big data) could help researchers to classify better the patient's molecular characteristics and drive clinicians to identify personalized therapeutic strategies. Here, Paolini et al. highlighted how the integration of “multiomic” data (i.e., miRNAs profiling and microbiota signature) with other omics (i.e., metabolomics, exposomics) analyzed by AI algorithms could improve the diagnostic and prognostic potential of specific biomarkers of disease.

Apart from the novel computational methods, Liu et al. proposed a new family of Trechisporales, Sistotremastraceae fam. Nov. based on the combination of molecular and morphological data, and it is typified by Sistotremastrum. The phylogenetic analyses show that Sistotremastraceae forms a monophyletic lineage with robust support within Trechisporales. Marine Streptomycetes are attracting particular attention as the new producer of novel antibiotics and anti-cancer agents with unusual properties. Shi et al. established the taxonomic status of a new Streptomycetes species isolated from a marine environment, and this new Streptomyces species shows a valuable source of new bioactive secondary metabolites. The great potential to produce novel natural products was evaluated by genomes analysis, compound detection, and antimicrobial activities. Rhoades et al. summarized several host factors and pathways involved in coronavirus infection and are also implicated in neuropsychiatric symptoms. Though several of these host factors are expressed in the central nervous system, they have also provided evidence that their influence on widespread systemic inflammation may play a significant role in the development of long-term psychological symptoms stemming from COVID-19 infection. Wang et al. went through the frontlines of the metaomics techniques and explored their potential applications in clinical diagnoses of human diseases, e.g., infectious diseases, through which we concluded that novel diagnostic methods based on human microbiomes should be achieved in the near future, while the limitations of these techniques such as standard procedures and computational challenges for rapid and accurate analysis of metaomics data in clinical settings were also examined.

Finally, we want to thank all the authors who contributed their original work to our special issue and the reviewers for their valuable comments. We would like to express our sincere gratitude to the editorial office of Frontiers in Microbiology for their excellent support and for providing us with this opportunity to successfully host this hot topic issue.

## Author contributions

QZ drafted the manuscript. GT revised the draft. Both authors made a direct and intellectual contribution to the work and approved the final version for publication.

## Funding

This study was supported by the Foundation of Education Department of Liaoning Province (Grant No. LJKZ0280).

## Conflict of interest

The authors declare that the research was conducted in the absence of any commercial or financial relationships that could be construed as a potential conflict of interest.

## Publisher's note

All claims expressed in this article are solely those of the authors and do not necessarily represent those of their affiliated organizations, or those of the publisher, the editors and the reviewers. Any product that may be evaluated in this article, or claim that may be made by its manufacturer, is not guaranteed or endorsed by the publisher.
